# Cross-cultural examination of the Big Five Personality Trait Short Questionnaire: Measurement invariance testing and associations with mental health

**DOI:** 10.1371/journal.pone.0226223

**Published:** 2019-12-17

**Authors:** Laura Mezquita, Adrian J. Bravo, Julien Morizot, Angelina Pilatti, Matthew R. Pearson, Manuel I. Ibáñez, Generós Ortet, Cross-Cultural Addictions Study Team

**Affiliations:** 1 Department of Basic and Clinical Psychology and Psychobiology, Universitat Jaume I, Castelló de la Plana, Castelló, Spain; 2 Centre for Biomedical Research Network on Mental Health (CIBERSAM), Instituto de Salud Carlos III, Castelló de la Plana, Castellón, Spain; 3 Department of Psychological Sciences, William & Mary, Williamsburg, Virginia, United States of America; 4 School of Psychoeducation, University of Montreal, Montreal, Quebec, Canada; 5 Facultad de Psicología, Universidad Nacional de Córdoba, Instituto de Investigaciones Psicológicas (IIPsi-UNC-CONICET), Córdoba, Córdoba, Argentina; 6 Center on Alcoholism, Substance Abuse, and Addictions, University of New Mexico, Albuquerque, New Mexico, United States of America; Aalborg University, DENMARK

## Abstract

The present study examined the measurement invariance of the Big Five Personality Trait Short Questionnaire (BFPTSQ) across language (Spanish and English), Spanish-speaking country of origin (Argentina and Spain) and gender groups (female and male). Evidence of criterion-related validity was examined via associations (i.e., correlations) between the BFPTSQ domains and a wide variety of mental health outcomes. College students (*n* = 2158) from the USA (*n* = 1117 [63.21% female]), Argentina (*n* = 353 [65.72% female]) and Spain (*n* = 688 [66.86% female]) completed an online survey. Of the tested models, an Exploratory Structural Equation Model (ESEM) fit the data best. Multigroup ESEM and ESEM-within-CFA generally supported the measurement invariance of the questionnaire across groups. Internalizing symptomatology, rumination and low happiness were related mainly to low emotional stability across countries, while low agreeableness and low conscientiousness were related chiefly to externalizing symptomology (i.e., antisocial behavior and drug outcomes). Some correlational differences arose across countries and are discussed. Our findings generally support the BFPTSQ as an adequate measure to assess the Big Five personality domains in Spanish- and English-speaking young adults.

## Introduction

According to a biopsychosocial model of psychopathology, several biological, psychological and social variables have been indicated to impact health outcomes [1]. One of the nonspecific distal psychological variables that influences psychopathology development is personality [2]. Personality traits have been associated with other outcomes, such as happiness [3], academic and job performance, antisocial and criminal conduct [4], and a broad spectrum of health-related behaviors [5,6].

The Five-Factor Model (FFM; a.k.a. Big Five) is one of the most widely accepted structural personality models [7]. The FFM proposes five broad personality traits: openness to experience, extraversion, agreeableness, conscientiousness, and neuroticism (or its positive pole, emotional stability). Openness represents individual differences in curiosity, fantasy, appreciation of art and beauty, and social attitudes. Extraversion reflects individual differences in sociability, social ascendency, activity, excitement seeking, and positive emotionality. Agreeableness reveals individual differences in compliance, empathy, collaboration, and altruism. Conscientiousness represents individual differences in being methodical, planning, impulse control, and respecting and abiding by conventional social norms and rules. Neuroticism refers to individual differences in the tendency to experience frequently and intensively negative emotions, such as anxiety, fear, depression and irritability, as well as having low self-esteem [8].

Traditionally, measures to assess personality traits encompass many items and are, therefore, time-consuming. Several shorter alternatives, including the Big Five Questionnaire-Children version [BFQ-C; 9], the Big Five Inventory [BFI; 7,10], the Ten-Item Personality Inventory [TIPI; 11], the Mini-International Personality Item Pool Big Five Measure [Mini-IPIP; 12], Mini Modular Markers [3M40; 13], the NEO Five-Factor Inventory-3 [NEO-FFI-3; 14], the short form of the Junior Spanish version of the NEO-PI-R [JS NEO-S; 15], and the Big Five Personality Trait Short Questionnaire [BFPTSQ; 8], have been developed. These brief versions are particularly useful when there is limited administration time and/or when the target population’s characteristics (e.g., adolescents, elderly) impede the use of full versions.

Among these short personality measures, the BFPTSQ [8] has a number of advantages. First, it has wider conceptual breadth (i.e., content validity) than most available measures, particularly very short ones. Indeed, many available short personality measures suffer from limited conceptual breadth, which essentially means that these measures do not represent a number of important lower-order or primary traits [see 16]. When developing the BFPTSQ, Morizot built it from the initial pool of the English BFI items [7,10], but added 8 new items to tap into important primary traits that were missing in the original short measures (e.g., sensation seeking, impulsiveness, openness to cultural differences, etc.). Further, 2 items that could generate confusion were deleted (“prefer work that is routine” and “generates a lot of enthusiasm”). Second, 36 items were reworded to be more easily understood than in the original BFI, so they may be utilized in assessments with both adolescents and adults (S1 Table presents the item correspondence between the BFI and the BFPTSQ). This is particularly important for long-term longitudinal studies because they often employ different measures for adolescents and adults depending on the participants’ ages at the time points when assessments are conducted. In such cases, determining whether differences are due to real changes in traits or to the measure taken to assess personality during different developmental periods is no easy task. Finally, this instrument is in the public domain, so it can be used freely by researchers for applied purposes.

Originally, the psychometric properties of the BFPTSQ were examined in French-speaking adolescents from Quebec [8]. The French BFPTSQ scores showed adequate psychometric properties, including evidence for content and structure validity and adequate internal consistency [8]. The BFPTSQ scores also correlated with the NEO-PI-3 [14], supporting its convergent validity. Finally, criterion validity was demonstrated by predicting psychopathology symptoms (i.e., conduct disorder, major depression disorder, attention deficit hyperactivity disorder, bipolar disorder, oppositional defiant disorder, social phobia, substance use and generalized anxiety disorder) and academic achievement (i.e., grade point average). Moreover, this version was found to be invariant across gender groups [8].

The Spanish BFPTSQ was adapted and validated in a sample of Spanish adults [17]. Findings supported not only the structure reported by Morizot [8], but also the criterion-related validity (e.g., correlations between emotional stability and extraversion with happiness, and low conscientiousness and extraversion with alcohol consumption). Notably, these are the only two studies that have examined the psychometric properties of the BFPTSQ. To our knowledge, no previous work has examined the adequacy of the English BFPTSQ version to assess personality traits in English-speaking populations to date. Interest in understanding human psychology and behaviors outside traditionally studied cultures has increasingly grown [i.e., Western populations; 18,19], particularly by conducting cross-cultural research. However, a first key step to conducting cross-cultural research is to demonstrate that a questionnaire works in similar ways (i.e., measurement invariance) across countries, languages or other groups (e.g., gender). Only when measurement invariance is met is it legitimate to make valid comparisons of results across groups. Lack of measurement equivalence can lead to biased conclusions being drawn about potential cross-cultural differences [20].

In addition, although there is currently an increasing demand for short scales, their construction is not exempt of difficulties [21]. Following recommendations proposed by Ziegler et al. [21], and taking into account that our research purpose is assessing the Big Five in college/university students from different countries, we explored the factorial validity of the BFPTSQ with rigorous statistical strategies: 1) a structural equation modeling (i.e., ESEM) approach, 2) a calculation of different internal consistency indices (rather than just Cronbach’s alpha), and 3) correlational analyses across groups examining the empirical evidence supporting the interpretation of the test scores (i.e., criterion validity).

Specifically, in the present study we: (a) test the BFPTSQ structure in two different Spanish-speaking countries (Argentina and Spain); b) test the structure of the English BFPTSQ version; (c) test the measurement invariance across countries (Argentina vs. Spain), languages (English vs. Spanish) and gender groups; (d) explore the internal consistency of the scales among groups; (e) examine the associations among the five BFPTSQ domains and a large number of psychological constructs (i.e., psychopathology, antisocial behavior, marijuana use and negative marijuana-related consequences, rumination and happiness) in college students from the USA, Argentina and Spain (i.e., criterion-related validity). We focused on this set of variables because substance use [22,23] and mental health problems [24–26] are particularly insidious among college students. Therefore, a valid measure like the BFPTSQ will facilitate the cross-cultural examination of personality traits and their associations with a large set of outcomes in college students from different cultures/countries. It will also be useful for identifying college students at more risk of developing substance-related and mental health problems.

## Method

### Participants and procedure

College students from one university in Spain, one university in Argentina, and two universities in the USA completed an online survey on personality traits, personal mental health and marijuana use behaviors [for more information see 27]. Although 2192 college students completed the BFPTSQ, only those cases with less than 5% of missing values were retained. After deleting these cases (*n* = 29), and the five cases who failed to report their gender, the final sample included 2158 undergraduate students. Table 1 presents the descriptive statistics of the three samples.

**Table 1:** Descriptive statistics.

	USA (63.21% female)		Argentina (65.72% female)		Spain (66.86% female)		USA-Arg	USA-Sp	Arg-Sp	Whole sample (64.78% female)	
	N	Mean (SD)	N	Mean (SD)	N	Mean (SD)	*d*	*d*	*d*	N	Mean (*SD*)
Age	1090	19.05 (2.27)	352	24.26 (5.46)	688	21.42 (3.97)	-1.24	-.73	.59	2130	20.68 (4.03)
Openness	1117	2.66 (.63)	353	2.97 (.67)	688	2.76 (.69)	-.48	-.15	.31	2158	2.74 (.66)
Extraversion	1117	2.55 (.78)	353	2.45 (.78)	688	2.54 (.74)	.13	.01	-.12	2158	2.53 (.77)
Agreeableness	1117	2.59 (.60)	353	2.71 (.59)	688	2.71 (.61)	-.20	-.20	.00	2158	2.66 (.60)
Conscientiousness	1117	2.44 (.63)	353	2.38 (.60)	688	2.34 (.66)	.10	.15	.06	2158	2.40 (.63)
Emotional stability	1117	1.97 (.74)	353	1.88 (.84)	688	2.03 (.78)	.11	-.09	-.19	2158	1.97 (.77)
Depression	1112	1.25 (1.06)	353	1.60 (1.03)	688	1.61 (.92)	-.33	-.36	-.01	2153	1.42 (1.02)
Anger	1111	1.17 (1.09)	353	1.39 (1.14)	688	1.36 (.99)	-.20	-.18	.03	2152	1.27 (1.07)
Mania	1112	.99 (.93)	353	1.08 (.87)	688	1.29 (.94)	-.10	-.32	-.23	2153	1.10 (.94)
Anxiety	1112	1.17 (1.03)	353	1.19 (.94)	688	1.06 (.83)	-.02	.12	.15	2153	1.14 (.95)
Somatic distress	1112	.78 (.96)	353	1.00 (1.00)	688	.87 (.87)	-.22	-.10	.14	2153	.84 (.94)
Suicidal ideation	1111	.39 (.88)	353	.38 (.91)	688	.32 (.79)	.01	.08	.07	2152	.37 (.86)
Psychosis	1112	.24 (.65)	353	.11 (.40)	688	.17 (.49)	.24	.12	-.13	2153	.20 (.57)
Sleep disturbance	1111	1.00 (1.18)	353	1.03 (1.23)	688	1.13 (1.15)	-.02	-.11	-.08	2152	1.05 (1.18)
Memory	1112	.61 (.97)	353	.59 (.95)	688	.53 (.87)	.02	.09	.07	2153	.58 (.93)
Repetitive thoughts	1112	.56 (.88)	353	.62 (.91)	688	.47 (.79)	-.07	.11	.18	2153	.54 (.86)
Dissociation	1110	.64 (1.02)	353	.63 (1.05)	688	.56 (.93)	.01	.08	.07	2151	.61 (1.00)
Personality functioning	1112	.96 (1.06)	353	1.01 (1.04)	688	.99 (1.00)	-.05	-.03	.02	2153	.98 (1.04)
Alcohol use	1106	.57 (.89)	353	.46 (.75)	688	.33 (.66)	.13	.31	.18	2147	.48 (.81)
Tobacco use	1109	.44 (.95)	353	.59 (1.23)	688	.63 (1.23)	-.14	-.17	-.03	2150	.53 (1.10)
Illicit drug use	1110	.39 (.89)	353	.36 (.79)	688	.25 (.65)	.04	.18	.15	2151	.34 (.81)
Total mental health score	1112	.79 (.68)	353	.86 (.56)	688	.83 (.51)	-.11	-.07	.06	2153	.82 (.61)
Antisocial behavior	1109	1.22 (.32)	346	1.24 (.20)	675	1.26 (.26)	-.07	-.14	-.09	2130	1.24 (.28)
Negative marijuana-related consequences	441	.16 (.20)	142	.17 (.17)	148	.21 (.19)	-.05	-.27	-.22	731	.17 (.19)
Marijuana frequency	1117	4.16 (8.26)	353	3.54 (7.22)	688	1.80 (5.61)	.08	.33	.27	2158	3.30 (7.41)
Marijuana quantity	444	7.16 (17.37)	142	6.09 (15.14)	148	7.41 (20.90)	.07	-.01	-.07	734	7.01 (17.73)
Rumination	1111	3.97 (1.31)	353	3.69 (1.41)	688	4.02 (1.28)	.21	-.04	-.25	2152	3.94 (1.32)
Happiness	1106	7.80 (2.02)	351	7.63 (1.90)	678	7.71 (1.66)	.09	.05	-.04	2135	7.75 (1.89)

Cohen’s *d* values of .20 .50 and .80 correspond to small, medium and large effect sizes, respectively [28].

Before the assessment of the participants, the ethic committee of the Universidad de Córdoba and Universitat Jaume I approved the study, as well as the Collaborative Institutional Training Initiative (CITI program) in the USA universities (ID: 21636999 and 21637000).

### Measures

At all the university sites, the participants were administered the questionnaires below.

#### Big Five personality traits

Personality traits were assessed with the 50-item *Big Five Personality Trait Short Questionnaire* [BFPTSQ; 8] at the US universities, and the Spanish version [17] at the sites in Argentina and Spain. This measure assesses the FFM personality traits on a 5-point Likert-type scale (0 = *Strongly Disagree*, 4 = *Strongly Agree*): *openness*, *extraversion*, *agreeableness*, *conscientiousness* and *emotional stability*. In the present study, all the reversed items were indicated with an *r* after the item number (e.g., 31r). Responses were summed on all five scales and divided by the number of their items [10]. Thus, the scale scores in the present study ranged from 0 to 4.

#### Mental health

Past 2-week psychopathology was assessed using the 23-item DSM-5 Self-Rated Level 1 Cross-Cutting Symptoms Measure-Adult [29]. For Spanish-speaking students, the Spanish version was administered [30]. Participants are asked, “During the past two weeks, how much (or how often) have you been bothered by the following problems?” and responded on a 5-point response scale (0 = *none*, *not at all*, 1 = *slightly or rarely*, *less than a day or two*; 2 = *mild*, *several days*; 3 = *moderately*, *more than half the days*, 4 = s*everely*, *nearly every day*). A score of 2 or higher in most domains, except substance use (score of 1 or higher), is suggestive of clinically-relevant mental health problems [31]. The measure has been validated with both clinical [31] and college students [26] samples.

The Cronbach’s alpha of the total scale in the current total sample was .92 (US: .94, Arg: .89, Sp: .88). In the case of the subscales with more than one item the Cronbach’s alphas were: depression .78 (US: .82, Arg: .77, Sp: .67), mania .55 (US: .65, Arg: .40, Sp: .45), anxiety .80 (US: .86, Arg: .74, Sp: .70), somatic distress .65 (US: .72, Arg: .62, Sp: .55), psychosis .83 (US: .86, Arg: .67, Sp: .78), repetitive thoughts and behaviors .76 (US: .77, Arg: .75, Sp: .75), and personality functioning .79 (US: .82, Arg: .75, Sp: .76).

The prevalence rates of potential symptom presentation for the 13 domains (for those domains with multiple items, percentages were averaged) of the DSM-5 Cross-Cutting Symptoms Measure in the whole sample were as follows: depression (34.60%), anger (37.17%), mania (25.40%), anxiety (24.06%), somatic distress (17.51%), suicidal ideation (10.50%), psychosis (4.60%), sleep disturbance (29.55%), memory problems (15.79%), repetitive thoughts and behaviors (12.22%), dissociation (16.60%), and personality functioning (22.48%). For substance use, the rates for specific substance are presented: alcohol use (31.72%), tobacco (24.14%), and illicit drug use (19.20%).

Significant differences in the prevalence rates of the following symptoms between countries were found: depression (US: 29.59%, Arg: 38.53%, Sp: 40.70%, χ^2^(2) = 26.06, *p* < .001), anger (US: 34.47%, Arg: 39.94%, Sp: 40.12%, χ^2^(2) = 7.18, *p* < .05), mania (US: 22.21%, Arg: 23.51%, Sp: 31.40%, χ^2^(2) = 19.70, *p* < .001), anxiety (US: 26.35%, Arg: 24.65%, Sp: 20.06%, χ^2^(2) = 19.70, *p* < .001), suicidal ideation (US: 12.15%, Arg: 12.15%, Sp: 7.99%, χ^2^(2) = 7.85, *p* < .05), psychosis (US: 6.56%, Arg: 1.98%, Sp: 2.76%, χ^2^(2) = 20.60, *p* < .001), memory problems (US: 17.90%, Arg: 14.45%, Sp: 13.08%, χ^2^(2) = 7.98, *p* < .05), alcohol use (US: 36.35%, Arg: 32.86%, Sp: 23.69%, χ^2^(2) = 31.62, *p* < .001), and illicit drug use (US: 20.00%, Arg: 22.66%, Sp: 16.13%, χ^2^(2) = 7.36, *p* < .05).

#### Antisocial behavior

Antisocial behavior was assessed with the Antisocial Behavior Scale [ABS; 32]. The ABS contains 35 items that describe various antisocial behaviors (i.e. “I have broken, ripped, or damaged public properties” or “I have used knives or sticks in fights”) on a 4-point response scale (1 = *Never or Almost Never*, 4 = *Very Frequently or Very Often*). Summing the responses to all the items provides a total score. A previous project undertaken by the research team translated the ABS into and adapted it to English. The preliminary results revealed that the scores for the Spanish and English ABS versions displayed good internal consistency. The various differential item functioning analysis indicated that items generally operate similarly across the three participating countries [33]. The Cronbach’s alpha of the scale in the current total sample was .93, and by country were: .95 US, .87 Argentina and .92 Spain.

#### Negative marijuana-related consequences

The 21-item B-MACQ was employed to assess negative marijuana-related consequences [34]. All the items scored dichotomously to reflect the absence/presence of any marijuana-related problem in the last month (0 = *no*, 1 = *yes*). The total score reflects all the consequences that individuals experienced in the last 30 days. Previous research supports the test-retest reliability, as well as the discriminant and convergent validity, of the B-MACQ [34], and has also measured invariance and criterion validity across cultures and languages [i.e., 27]. The Cronbach’s alpha of the scale in the current total sample was .87, and by country were: .89 US, .81 Argentina and .86 Spain.

#### Marijuana use

Frequency of marijuana use was assessed by this question: “How many days in the last 30 days have you used marijuana?” If the participants responded 1 or higher, they completed the marijuana quantity measure. To report the consumed amount of marijuana, the participants were administered a visual guide indicating several amounts of marijuana in grams. Their typical weekly marijuana use in the last 30 days was assessed by the Marijuana Use Grid [MUG; 35]. The participants were asked to estimate the amount of marijuana they used in grams during each 4-hour period per day of a typical week. By adding all the values, an estimate of the typical amounts of marijuana used was made, which reflected the total grams marijuana they used in a typical week.

#### Rumination

Rumination was measured by the Ruminative Thought Style Questionnaire [RTSQ; 36]. The participants were asked to express how well each item described them on a 7-point response scale (1 = *Not at all*, 7 = *Very Well*). In Argentina and Spain, the Spanish RTSQ version was utilized [see the translating and adaptation procedures in 37]. According to the former findings obtained with the USA, Argentinian and Spanish samples, a 15-item version of this measure was employed, which proved invariant across genders and countries [37]. The Cronbach’s alpha of the scale in the current total sample was .94, and by country were: .95 US, .94 Argentina and .94 Spain.

#### Happiness

One question was about general happiness. The participants had to respond about how happy they felt in general that day (by attempting to ignore any feelings they had yesterday) on a 10-point scale (1 = *Completely Unhappy* to 10 = *Completely Happy*).

### Statistical analysis

All the analyses were done with version 25 of SPSS and version 7.4 of M*plus* [38]. The robust maximum likelihood estimator (MLR) was used in each analysis conducted in M*plus*. The MLR provides adjusted standard errors and statistical fit tests that are robust to data non-normality. The 99% confidence intervals (CI) of the relevant estimates were calculated and reported. Two model types were employed to assess factor validity: the ICM-CFA (independent clusters model confirmatory factor analysis) and the ESEM (exploratory structural equation modeling) with target loading rotation.

In line with Marsh et al. [39] and Morizot [8], all the factor models were estimated both with and without *a priori* correlated uniquenesses, employed to reflect that some items relate to the same primary trait or subdomain, and they share either a similar content, but reversed scores, or contain the same word. Twenty-seven *a priori* correlated uniquenesses were posited. Specifically, the correlated uniquenesses introduced for openness were: 1 with 21, 11 with 36, 16 with 21, 26 with 41r, 26 with 46, 1 with 16, 41r with 46; for extraversion: 7r with 32r, 2 with 22r, 12 with 42, 2 with 27, 17 with 27; for agreeableness: 18 with 23, 8 with 33, 23 with 33, 23 with 43, 18 with 43; for conscientiousness: 29 with 39, 19r with 24r, 19r with 39, 9r with 19r, 4 with 14; and for emotional stability: 10 with 35, 10 with 15r, 5r with 25, 5r with 45r, 30r with 50r. A detailed description of the conducted ESEM and ICM-CFA models can be found in Morizot [8].

The model fit assessment was made according to various indices [40]. The chi-square test was run for all the models. Although a nonsignificant chi-square indicates a good fitting model, this test is generally too sensitive with large sample sizes. Thus, other fit indices were calculated. Values of .08 or lower for the root mean square error of approximation (RMSEA), values of .90 or more for the comparative fit index (CFI) and Tucker–Lewis index (TLI) and values of .10 or less for the standardized root mean square residual (SRMR) suggest acceptable model fit [41,42]. For the RMSEA 90% CI values, those under .05 for the lower bound and under .08 for the upper bound indicate acceptable fit [43].

After identifying the best factor model, factor structure was tested in each country, and measurement invariance was tested between the Spanish-speaking groups (Argentina and Spain), the Spanish and English versions, and across gender groups using multi-group ESEM. These models were assessed with a series of increasingly stringent multiple-group models (see [8]): configural invariance (MG1; all the loadings, intercepts and uniquenesses are freely estimated, with latent variances being constrained to 1 and latent means to 0), metric invariance (MG2; loadings constrained to invariance to make free estimations of the factor variances in one group), scalar invariance (MG3; intercepts constrained to invariance, to make free estimations of the factor means in one group), strict invariance (MG4; uniquenesses constrained to equality), correlated uniquenesses invariance (MG5), variance/covariance invariance (MG6; they must all be done simultaneously in ESEM), and latent means invariance (MG7). For all the models in this sequence, the imposed constraints are additive and the preceding model acts as a reference.

If there is evidence for noninvariance of the factor loadings across groups, as partial factor loading invariance cannot be tested in ESEM [44,45], an ESEM-within-CFA (ES-W-C) multi-group model was utilized. For the ES-W-C model, all parameter estimates from the ESEM solution were used as starting values. In addition, we added a total of 25 constraints (the square of the number of factors) to the ES-W-C model so that it was identified. Specifically, the 5 factor variances for the first group of the multiple group solution and the 20 “anchor items” were fixed. The anchor item or referent indicator for each factor is the item that has a large loading for the factor that it is designed to measure and small cross-loadings on other factors. Then these small cross-loadings were fixed to their values from the ESEM solution. This allowed a higher level of convergence with the ESEM solution. For all other parameter estimates, the patterns of the fixed and free estimates were the same as in the selected ESEM solution [44]. It is noteworthy that, in ES-W-C, the factor variances were fixed to one in the first group to identify the model. Then the covariances invariance across groups was tested, rather than the variance/covariance invariance.

To assess changes in the model fit tests, the Satorra-Bentler scaled chi-square test [46] was computed. However, the chi-square difference test is sensitive to sample size [47]. For this reason, more comparisons in the increment of other indices were made to test the invariance between less and more constrained models. In order to consider a model to be invariant, the ΔCFI should be ≤.010 and the ΔRMSEA should be ≤ .015 [48,49].

In both the Spanish and English questionnaire versions, sources of reliability were explored by resorting to Cronbach’s alphas and ordinal omegas [50]. The sources of evidence for criterion validity were explored with Pearson correlations among all the personality dimensions and psychopathology, antisocial behavior, marijuana outcomes, rumination and happiness in all three countries.

## Results

### Descriptive analysis

Table 1 provides the descriptive statistics (means/standard deviations) for all the personality dimensions, criterion variables and the participants’ ages for the whole sample and per country. The comparison made of the magnitude of the mean differences across countries indicated that, despite medium (USA and Spain; Spain and Argentina) and large (USA and Argentina) differences in the participants’ ages across countries, all the differences in personality and the criterion variables were small (all the *ds* were below .50).

### Factor structure

When studying the BFPTSQ structure in the total sample, the best fitting model was the ESEM model, in which correlated uniquenesses were allowed (M2b). See the fit indices of all the models performed in Table 2. Table 3 reports the standardized factor loadings for the whole sample. All the items had significant factor loadings on its hypothesized factor, except for item 31r (“Is not really interested in different cultures, their customs and values”) and 41r (“has few artistic interests”) on the openness factor. All the items for the conscientiousness factor and emotional stability presented the highest factor loading on their intended factor. Eight extraversion factor items showed the highest factor loadings on its hypothesized factor, while items 42 (“likes exciting activities that provide thrills”) and 47 (“has a tendency to laugh and have fun easily”) showed the highest factor loadings on the openness factor. Five agreeableness factor items had the highest factor loadings on its intended factor (items 3r, 13r, 28r, 38r, 48r), and five items showed similar cross-loadings between agreeableness and the openness factor (items 8, 18, 23, 33, 43). Table 4 shows the latent factor correlations from the final ESEM and the ICM-CFA.

**Table 2:** Goodness-of-fit statistics from the confirmatory factor analytic, Exploratory Structural Equation Models (ESEM) and ESEM-Within-CFA (EWC) Models.

	χ^2^_S-B_ (df)	CFI	TLI	RMSEA	90% CI	SRMR	Ref	Δχ^2^_S-B_ (df)	ΔCFI	ΔRMSEA
M1: ICM-CFA	16359.183*** (1165)	.598	.577	.078	.077 .079	.110	-	-	-	-
M1b: ICM-CFA with CUs	13226.266*** (1138)	.680	.656	.070	.069 .071	.110	M1	2560.487*** (27)	.082	-.008
M2: ESEM	7715.190*** (985)	.822	.778	.056	.055 .057	.041	M1	8249.826*** (180)	.224	-.022
M2b: ESEM with CUs	4562.264*** (958)	.905	.878	.042	.041 .043	.034	M2	7471.147*** (27)	.083	-.014
M2b: Argentina	1678.284*** (958)	.887	.855	.046	.042 .050	.042	-	-	-	-
M2b: Spain	2042.032*** (958)	.914	.890	.041	.038 .043	.038	-	-	-	-
M2b: USA / English version	3065.349*** (958)	.900	.872	.044	.043 .046	.034	-	-	-	-
**Invariance among Spanish speakers (Argentina vs Spain)**										
MG1: Configural invariance	3730.054*** (1916)	.905	.878	.043	.041 .045	.039	-	-	-	-
MG2: λ invariant	3978.289*** (2141)	.904	.890	.041	.039 .043	.047	M1	248.235 (225)	-.001	-.002
MG3: λ, τ invariant	4348.732*** (2186)	.887	.873	.044	.042 .045	.049	M2	370.443*** (45)	-.017	.003
MG3b: λ, τ partially invariant	4194.487*** (2182)	.894	.882	.042	.040 .044	.048	M2	216.198*** (41)	-.010	.001
MG4: λ, τ, δ invariant	4272.918*** (2232)	.893	.883	.042	.040 .044	.050	M3b	78.431** (50)	-.001	.000
MG5: λ, τ, δ, CUs invariant	4297.314*** (2259)	.893	.884	.042	.040 .044	.050	M4	670.357*** (27)	.000	.000
MG6: λ, τ, δ, CUs, ξ/φ invariant	4318.434*** (2274)	.893	.885	.042	.040 .043	.055	M5	25.959* (15)	.000	.000
MG7: λ, τ, δ, CUs, ξ/φ, η invariant	4342.998*** (2279)	.892	.884	.042	.040 .044	.057	M6	24.564*** (5)	-.001	.000
**Invariance among English vs. Spanish**										
MG1: Configural invariance	5676.818*** (1916)	.905	.878	.043	.041 .044	.035	-	-	-	-
MG2: λ invariant	6531.508*** (2141)	.889	.873	.044	.042 .045	.048	M1	850.331*** (225)	-.016	.001
MG2b: λ partially invariant	6287.323*** (2135)	.895	.879	.042	.041 .044	.044	M1	611.473*** (219)	-.010	-.001
MG3: λ, τ invariant	7303.637*** (2180)	.870	.854	.047	.045 .048	.047	M2b	806.199*** (45)	-.025	.005
MG3b: λ, τ partially invariant	6713.462*** (2172)	.885	.870	.044	.043 .045	.045	M2b	457.729*** (37)	-.010	.002
MG4: λ, τ, CUs invariant	6908.488*** (2199)	.881	.867	.045	.043 .046	.047	M3b	180.723*** (27)	-.004	.001
MG5: λ, τ, CUs, δ invariant	7308.848*** (2249)	.872	.860	.046	.044 .047	.051	M4	347.485***(50)	-.009	.001
MG6: λ, τ, CUs, δ, φ invariant	7377.278*** (2259)	.870	.859	.046	.045 .047	.056	M5	68.430*** (10)	-.002	.000
MG7: λ, τ, CUs, δ, φ, η invariant	7622.050*** (2264)	.864	.853	.047	.046 .048	.058	M6	521.487*** (5)	-.006	.001
**Gender invariance**										
MG1: Configural invariance	5605.712*** (1916)	.902	.875	.042	.041 .044	.036	-	-	-	-
MG2: λ invariant	5997.169*** (2141)	.898	.883	.041	.040 .042	.042	M1	395.338** (225)	-.004	-.001
MG3: λ, τ invariant	6184.850*** (2186)	.894	.881	.041	.040 .042	.043	M2	193.842** (45)	-.004	.000
MG4: λ, τ, δ invariant	6417.212*** (2236)	.889	.879	.042	.040 .043	.048	M3	222.814** (50)	-.005	.001
MG5: λ, τ, δ, CUs invariant	6434.418*** (2263)	.890	.880	.041	.040 .043	.048	M4	33.978 (27)	.001	-.001
MG6: λ, τ, δ, CUs, ξ/φ invariant	6513.279*** (2278)	.888	.879	.041	.040 .043	.056	M5	75.451** (15)	-.002	.000
MG7: λ, τ, δ, CUs, ξ/φ, η invariant	6835.929*** (2283)	.879	.871	.043	.042 .044	.062	M6	397.970** (5)	-.009	.002

ICM-CFA = independent clusters model confirmatory factor analysis; ESEM = exploratory structural equation modeling; χ2 = chi square; df = degrees of freedom; CFI = comparative fit index; TLI = Tucker–Lewis index; RMSEA = root mean square error of approximation; 90% CI = 90% confidence interval of the RMSEA; SRMR = standardized root mean square residual; Ref = reference model; ΔSχ2 = Satorra–Bentler scaled chi-square difference test; Δdf = change in degrees of freedom; ΔCFI = change in CFI; ΔRMSEA = change in RMSEA; λ = factor loadings; τ = intercepts; CUs = correlated uniqueness; δ = uniquenesses; ξ = factor variances; φ = factor covariances; η = factor means.

*p < .05

**p < .01

***p < .001.

**Table 3:** Standardized factor loadings from the exploratory structural equation model of the BFPTSQ in the whole sample (M2b) and in the English and Spanish versions of the questionnaire (MG1).

	λ Openness			λ Extraversion			λ Agreeableness			λ Conscientiousness			λ Emotional stability			δ	δ	δ
Item	Whole sample	English	Spanish	Whole sample	English	Spanish	Whole sample	English	Spanish	Whole sample	English	Spanish	Whole sample	English	Spanish	Whole sample	English	Spanish
1	**.411**	**.345**	**.460**	**.254**	**.253**	**.254**	.036	.020	.024	**.146**	**.264**	**.090**	.046	-.020	.084	.638	.610	.629
6	**.501**	**.440**	**.438**	**.116**	**.115**	**.093**	**.180**	.096	**.224**	**.088**	**.241**	.023	**-.078**	**-.126**	-.059	.632	.597	.687
11	**.434**	**.400**	**.535**	.047	-.014	**.101**	**.090**	.031	.051	**.086**	**.131**	**.130**	**.082**	.048	**.110**	.748	.798	.603
16	**.480**	**.481**	**.473**	**.151**	**.187**	**.128**	.066	**.139**	.043	.018	.018	-.002	-.028	**-.100**	.019	.704	.648	.725
21	**.461**	**.458**	**.475**	**.162**	**.185**	**.151**	**.077**	**.136**	.034	.037	.036	.040	.018	-.048	.059	.706	.676	.699
26	**.430**	**.414**	**.383**	.053	**.099**	.021	**.151**	**.212**	**.123**	.001	.002	-.010	-.050	**-.181**	.047	.776	.714	.812
31r	.062	-.065	.066	**.082**	.088	.056	**.373**	**.348**	**.239**	.000	.104	-.012	**-.081**	**-.150**	-.002	.851	.836	.926
36	**.453**	**.458**	**.426**	**.064**	**.094**	.031	**.132**	.092	**.171**	**.213**	**.273**	**.167**	.044	.047	.038	.637	.576	.684
41r	.053	-.061	**.147**	.033	.017	.028	**.237**	**.166**	.103	-.010	.064	.034	.023	-.010	.080	.936	.960	.942
46	**.297**	**.325**	**.290**	.027	.013	.045	.034	.045	.024	.011	-.033	.052	-.027	-.057	-.005	.903	.887	.898
2	**.194**	.103	**.212**	**.572**	**.580**	**.553**	.029	-.072	.080	**.075**	**.181**	.047	**-.073**	**-.109**	-.044	.563	.529	.573
7r	**-.280**	**-.350**	**-.184**	**.829**	**.829**	**.821**	.024	-.001	.015	**-.097**	**-.145**	-.035	**.072**	**.127**	.047	.319	.298	.336
12	**.241**	**.280**	**.217**	**.355**	**.370**	**.347**	.007	.030	.099	**.166**	.110	**.189**	**.157**	**.195**	**.096**	.642	.621	.663
17	**.234**	**.200**	**.389**	**.389**	**.423**	**.401**	**-.225**	**-.099**	**-.248**	**.256**	**.292**	**.166**	**.079**	.044	**.105**	.581	.547	.574
22r	**-.331**	**-.410**	**-.283**	**.892**	**.885**	**.877**	**.092**	.058	.062	**-.072**	-.090	-.021	**-.059**	-.030	-.040	.241	.253	.244
27	**.264**	**.269**	**.362**	**.359**	**.426**	**.326**	**-.161**	**-.125**	**-.118**	**.198**	**.160**	**.195**	**.269**	**.240**	**.281**	.543	.524	.550
32r	**-.282**	**-.353**	**-.209**	**.854**	**.875**	**.836**	.028	.045	-.025	**-.062**	-.084	-.020	**.053**	**.083**	.056	.281	.238	.311
37	**.067**	.047	**.077**	**.813**	**.839**	**.793**	-.028	-.011	.045	**-.062**	-.116	-.053	.008	.038	-.028	.339	.320	.350
42	**.377**	**.446**	**.296**	**.270**	**.332**	**.234**	-.081	.035	.002	-.063	-.055	**-.165**	.027	.041	-.015	.755	.641	.830
47	**.400**	**.401**	**.235**	**.342**	**.399**	**.298**	**.184**	**.212**	**.311**	.069	**.117**	-.030	.029	-.047	.060	.576	.470	.669
3r	-.065	-.048	**-.125**	**-.132**	**-.122**	**-.131**	**.438**	**.563**	**.340**	-.056	**-.189**	.030	**.239**	**.231**	**.256**	.716	.607	.774
8	**.439**	**.341**	**.310**	**.134**	**.173**	**.076**	**.333**	**.316**	**.552**	**.179**	**.280**	.029	**-.113**	**-.173**	**-.115**	.541	.498	.511
13r	.012	-.024	-.076	**-.118**	**-.111**	**-.135**	**.574**	**.560**	**.486**	**.106**	**.094**	**.164**	**.090**	.026	**.182**	.601	.638	.630
18	**.390**	**.467**	**.189**	.044	.084	.024	**.257**	**.362**	**.368**	-.056	-.104	-.090	**.087**	.045	.067	.770	.668	.799
23	**.317**	**.346**	**.162**	**.133**	**.121**	**.136**	**.269**	**.293**	**.411**	**-.109**	-.117	**-.162**	.057	.080	-.006	.803	.790	.761
28r	**-.239**	**-.231**	**-.370**	**.247**	**.153**	**.340**	**.431**	**.568**	**.401**	-.007	-.033	-.031	**.080**	**.155**	.024	.681	.551	.647
33	**.400**	**.388**	**.203**	**.088**	**.113**	.067	**.369**	**.419**	**.552**	**.182**	**.235**	.028	-.042	**-.112**	-.018	.566	.454	.582
38r	-.075	-.129	**-.172**	**-.139**	**-.164**	**-.136**	**.651**	**.669**	**.534**	**.088**	**.106**	**.134**	**.132**	**.123**	**.168**	.484	.430	.598
43	**.377**	**.372**	**.237**	**.231**	**.304**	**.151**	**.333**	**.354**	**.496**	.082	.051	.016	-.053	-.050	**-.087**	.604	.550	.603
48r	**-.187**	**-.239**	**-.331**	**-.087**	**-.134**	-.069	**.541**	**.463**	**.537**	**.084**	**.118**	**.122**	-.016	-.023	.003	.654	.682	.631
4	**.239**	**.172**	**.316**	**.050**	**.098**	-.007	.053	-.040	.070	**.552**	**.631**	**.548**	**-.095**	**-.089**	**-.104**	.554	.478	.547
9r	**-.377**	**-.320**	**-.295**	-.044	-.050	.004	**.102**	**.305**	-.064	**.531**	**.345**	**.624**	.009	**.110**	-.034	.634	.636	.585
14	**.251**	**.182**	**.267**	**.086**	**.112**	.044	**.171**	**.122**	**.235**	**.482**	**.579**	**.393**	**-.109**	**-.074**	**-.150**	.555	.458	.627
19r	**-.384**	**-.416**	**-.229**	-.028	.006	-.051	.082	.081	-.003	**.506**	**.383**	**.616**	.003	**.090**	-.029	.662	.705	.614
24r	**-.353**	**-.390**	**-.210**	**.106**	**.086**	**.136**	.022	.022	-.024	**.502**	**.466**	**.521**	**.091**	**.167**	.069	.633	.623	.656
29	**.144**	**.217**	**.159**	-.031	.007	-.032	**-.134**	-.101	-.005	**.603**	**.569**	**.537**	-.032	.016	**-.083**	.618	.586	.689
34	**.237**	**.252**	**.298**	.047	.034	**.076**	-.016	-.013	.081	**.575**	**.558**	**.499**	.023	**.104**	-.044	.527	.518	.571
39	.084	.111	**.154**	.012	.062	-.003	**-.132**	-.079	-.055	**.650**	**.609**	**.580**	**-.071**	-.022	**-.111**	.583	.576	.654
44r	**-.372**	**-.411**	**-.190**	-.024	**-.096**	.063	-.028	-.026	**-.134**	**.497**	**.514**	**.514**	**.205**	**.254**	**.203**	.612	.566	.639
49r	**-.215**	**-.352**	**-.144**	**-.203**	**-.210**	**-.206**	**.317**	**.273**	**.226**	**.352**	**.470**	**.303**	**.161**	.076	**.272**	.637	.552	.681
5r	**-.088**	-.040	**-.087**	**.161**	**.135**	**.195**	.053	.024	.062	**.097**	**.138**	**.092**	**.544**	**.577**	**.524**	.575	.555	.591
10	**.378**	**.418**	**.343**	-.027	.012	-.046	**-.114**	-.084	-.011	-.047	.089	**-.183**	**.662**	**.606**	**.656**	.494	.500	.511
15r	**-.112**	-.059	**-.113**	.007	-.011	.028	.035	-.008	.040	**-.182**	**-.185**	**-.115**	**.672**	**.707**	**.636**	.538	.484	.587
20r	**-.225**	**-.124**	**-.219**	.049	.067	.036	**-.181**	**-.087**	**-.242**	**-.090**	-.045	**-.191**	**.564**	**.670**	**.514**	.616	.508	.639
25	**.275**	**.344**	**.229**	-.007	.017	.004	-.055	.000	.043	**.081**	.109	.014	**.593**	**.520**	**.623**	.566	.610	.537
30r	**-.129**	-.100	**-.151**	-.036	**-.074**	.012	**.261**	**.278**	**.151**	.007	.006	.075	**.579**	**.555**	**.632**	.517	.547	.484
35	**.386**	**.469**	**.348**	-.046	.013	-.069	**-.110**	-.047	.024	**.139**	**.189**	.006	**.560**	**.513**	**.577**	.521	.484	.555
40r	**-.123**	**-.169**	-.038	**.122**	**.254**	.009	-.001	-.060	-.024	-.066	-.048	-.013	**.684**	**.621**	**.751**	.483	.470	.444
45r	**-.162**	**-.205**	.001	**.213**	**.173**	**.267**	-.035	-.018	**-.159**	**.177**	**.224**	**.190**	**.458**	**.451**	**.491**	.613	.607	.583
50r	-.017	-.019	-.070	**-.091**	**-.106**	-.065	**.392**	**.431**	**.258**	.022	.047	.075	**.551**	**.452**	**.653**	.464	.539	.408

The column “whole sample” presents the factor loadings of the M2b model, while the columns “English” and “Spanish” correspond to the factor loadings of the MG1 model of the Invariance between the English vs. Spanish versions. The fit indices of both models are presented in Table 2. Bold denotes all the significant factor loadings (the 99% CI does not cross zero). λ = factor loadings; δ = uniquenesses.

**Table 4:** Correlations between personality factors.

	Openness	Extraversion	Agreeableness	Conscientiousness	Emotional Stability
Openness	-	**.203**	.025	**.231**	-.055
Extraversion	**.297**	-	.070	**.286**	**.217**
Agreeableness	**.453**	**.285**	-	**.262**	**.200**
Conscientiousness	**.358**	**.281**	**.529**	-	**.248**
Emotional Stability	**.103**	**.338**	**.266**	**.277**	-

Latent factor correlations from the final exploratory structural equation model (ESEM) are presented above the diagonal line, while latent correlations form the independent clusters confirmatory factor analysis (ICM-CFA) are presented below the diagonal line. Bold denotes all the significant correlations (the 99% CI does not cross zero).

Upon finding the best factor solution, an ESEM was performed in each country. Fit indices were acceptable for the Spanish sample. In the Argentinian sample, the CFI and TLI were close, but lower than .90. However, the RMSEA, RMSEA 90% CI values and SRMR were adequate (≤.05). In both samples, factor loadings were salient and significant in its hypothesized factor, except for items 31 and 41 in the openness to the experience factor (see S2 Table). All the items for conscientiousness, agreeableness and emotional stability presented the highest factor loading on their intended factor, while items 27 (“show self-confidence, is able to assert himself/herself”) and 42 (“likes exciting activities that provide thrills”) from the extraversion factor showed similar cross-loadings in the extraversion and openness to the experience factor.

The fit indices of the English version were adequate. The factor loadings of the English version are presented in Table 3 (i.e., as they are the same as those obtained in the configural invariance model across the English and Spanish versions), and they were very similar to those found in the whole sample.

### Measurement invariance

A few minor differences emerged across groups when studying the invariance of the Spanish questionnaire version between the Argentinian and Spanish participants. Constraining the intercepts across Spanish speakers resulted in a ΔRMSEA below .015, and ΔCFI was -.017 (MG3). Hence a model with a partial invariance of intercepts (MG3b) was estimated. Based on the modification indices, four items across groups were freed: 13r (“provokes quarrels or arguments with others”), 38r (“can sometimes be rude or mean to others”); 43 (“likes to cooperate with others”) (Arg > Sp) from the agreeableness factor; 49r (“can do things impulsively without thinking about the consequences”) (Arg > Sp) from the conscientiousness factor. This model gave a better fit than the model with the fully invariant intercepts and ΔCFI ≤ .01. When further constraints were included (MG4 to MG7), ΔCFI was ≤ .01 and ΔRMSEA was ≤ .015, which suggested reasonable invariance across groups. Considering that the structure of the Spanish BFPTSQ had been previously studied [17], and small differences across Spanish-speaking samples had also been found, the Argentinian and Spanish samples were considered together when the structure of the Spanish version was compared with the English version.

To test the measurement invariance of the English and Spanish (Spanish and Argentinian combined samples) versions, a configural invariance model was performed (MG1). This model showed acceptable fit indices as it can be seen in Table 2. Its factor loadings are presented in Table 3. When the factor loadings were constrained across Spanish and English speakers, ΔRMSEA was .001 and ΔCFI was -.016 (MG2). Therefore, an ES-W-C was run to test the partial metric invariance (MG2b). According to the modification indices, six factor loadings (6 of 250) were freely estimated across groups. One was a difference in the nonstandardized factor loadings of one item on their target factor: 20r (“worries a lot about many things”) on the emotional stability factor (Spanish = .454 [.361 .546]; English = .772 [.668 .876]). The others were differences in cross-loadings. Adding constraints between the intercepts across groups also indicated differences (MG3, ΔCFI = -.025). Thus, a model with partial invariance of intercepts (MG3b) was estimated. According to the modification indices, eight items were freed across groups: 4 (“works conscientiously, does the things he/she has to do well”) (Eng < Sp); 9r (“can be a little careless and negligent”) (Eng > Sp); 11 (“Is ingenious, reflects a lot”) (Eng < Sp); 20r (“worries a lot about many things”) (Eng > Sp); 22r (“is rather quiet, does not talk much”) (Eng > Sp); 28r (“can be distant and cold with others”) (Eng > Sp); 32r (“is timid, shy”) (Eng > Sp); 36 (“likes to reflect, tries to understand complex things”) (Eng < Sp). This model indicated a better fit than the model with the fully invariant intercepts and gave ΔCFI ≤ .01. Including additional constraints (MG4-MG7) gave ΔRMSEA ≤ .015 and ΔCFI ≤ .01, which suggests invariance across groups. Note that for convergence problems, in the case of invariance between the English vs. the Spanish version, the correlated uniquenessess invariance (MG4) was tested first followed by the measurement errors invariance (MG5), rather than backwards.

The results of the invariance analyses done across gender are also presented in Table 2 and indicated that this model was completely invariant (all the ΔCFI ≤ .01, and the ΔRMSEA ≤ .015) when specifying the constraints among factor loadings (MG2), intercepts (MG3), measurement errors (MG4), correlated uniqueness (MG5), variances and covariances (MG6) and factor means (MG7) across groups of males and females.

### Internal consistency

Table 5 shows the internal consistency indices. Cronbach’s alpha and the ordinal omega of all the scales were .70 or higher, except for the ordinal omega of the agreeableness scale in the English version, which came close to the recommended cut-off of .70 (i.e., .689 [.617 .754]).

**Table 5:** Internal consistencies of the scales.

	Whole sample		English		Spanish	
	α 99%CI	Ω 99%CI	α 99%CI	Ω 99%CI	α 99%CI	Ω 99%CI
Openness	.820 [.805 .835]	.816 [.796 .832]	.801 [.778 .824]	.804 [.778 .825]	.835 [.815 .854]	.821 [.789 .845]
Extraversion	.848 [.835 .861]	.868 [.854 .879]	.859 [.842 .875]	.879 [.863 .893]	.840 [.820 .859]	.860 [.840 .876]
Agreeableness	.757 [.736 .776]	.723 [.679 .758]	.763 [.734 .789]	.689 [.617 .754]	.754 [.723 .782]	.744 [.701 .777]
Conscientiousness	.789 [.772 .806]	.767 [.725 .796]	.790 [.765 .814]	.748 [.689 .795]	.790 [.764 .814]	.788 [.742 .816]
Emotional stability	.852 [.839 .864]	.853 [.839 .864]	.845 [.827 .863]	.846 [.825 .865]	.862 [.845 .878]	.866 [.846 .882]

α = Cronbach’s alphas, Ω = Ordinal omega

### Criterion-related validity

The correlations between personality domains and criterion variables in all three countries are presented in Table 6. The results demonstrated that health outcomes were related to low emotional stability, low agreeableness, low conscientiousness and low extraversion. Internalizing symptomatology (i.e., depression, anxiety and somatic distress) showed the closest associations with low emotional stability in all three countries. Some externalizing behaviors were related to low agreeableness and low conscientiousness in the three countries (e.g., antisocial behavior), and others in only USA and Spain (e.g., alcohol, tobacco and illicit drug use). The correlations found for personality dimensions and the marijuana-related variables were low, but most of the significant associations were found with low conscientiousness. Finally, rumination in the three countries was related mainly to low emotional stability, and also to low conscientiousness, low agreeableness and low extraversion, but to a lesser extent. Happiness correlated mainly with emotional stability, followed by extraversion.

**Table 6:** Correlations between the five dimensions of the BFPTSQ and the criterion variables.

	Openness			Extraversion			Agreeableness			Conscientiousness			Emotional Stability		
	USA	Arg	Sp	USA	Arg	Sp	USA	Arg	Sp	USA	Arg	Sp	USA	Arg	Sp
Depression	-.030	-.026	-.007	-.296***	-.253***	-.237***	-.195***	-.134*	-.145***	-.363***	-.255***	-.247***	-.538***	-.541***	-.397***
Anger	-.068*	-.065	-.112**	-.217***	-.226***	-.113**	-.224***	-.148**	-.216***	-.253***	-.110*	-.038	-.473***	-.480***	-.428***
Mania	-.004	.138**	.082*	-.038	.098	.079*	-.146***	-.024	.015	-.208***	-.004	.049	-.151***	-.058	-.048
Anxiety	-.018	.023	-.074	-.342***	-.198***	-.170***	-.165***	-.209***	-.195***	-.305***	-.117*	-.157***	-.576***	-.563***	-.433***
Somatic distress	-.029	.005	-.071	-.203***	-.033	-.100**	-.169***	-.181***	-.147***	-.244***	-.104	-.047	-.390***	-.422***	-.279***
Suicidal ideation	-.117***	.023	-.106**	-.259***	-.141**	-.168***	-.193***	-.110*	-.139***	-.252***	.061	-.141***	-.282***	-.345***	-.232***
Psychosis	-.251***	-.103	-.104**	-.208***	-.099	-.097*	-.241***	-.128*	-.220***	-.223***	-.058	-.130***	-.122***	-.057	-.091*
Sleep disturbance	-.029	-.039	-.042	-.129***	-.193***	-.147***	-.104***	-.137*	-.123**	-.232***	-.148**	-.070	-.367***	-.365***	-.329***
Memory	-.095*	-.050	-.056	-.160***	-.125*	-.103**	-.219***	-.105*	-.141***	-.355***	-.160**	-.205***	-.278***	-.242***	-.158***
Repetitive thoughts	-.120***	.039	-.087*	-.213***	-.204***	-.168***	-.242***	-.185***	-.229***	-.287***	-.158**	-.222***	-.340***	-.394***	-.288***
Dissociation	-.070*	.003	-.095*	-.213***	-.207***	-.178***	-.192***	-.123*	-.196***	-.292***	-.164**	-.175***	-.362***	-.375***	-.294***
Personality functioning	-.019	-.033	-.030	-.318***	-.325***	-.226***	-.181***	-.214***	-.222***	-.347***	-.253***	-.221***	-.464***	-.512***	-.378***
Alcohol use	-.130***	-.025	-.076*	.014	.006	-.104**	-.174***	-.044	-.196***	-.219***	-.032	-.216***	-.040	.076	-.021
Tobacco use	-.084**	.026	.027	-.046	.062	.104**	-.187***	.080	-.126***	-.240***	-.066	-.103**	-.078**	.005	-.074
Illicit drug use	-.030	-.060	-.061	-.064*	.040	-.064	-.129***	-.014	-.145***	-.195***	-.086	-.127***	-.065*	-.042	-.119**
Total mental health score	-.087**	.001	-.077*	-.292***	-.227***	-.200***	-.259***	-.214***	-.269***	-.393***	-.216***	-.229***	-.494***	-.571***	-.449***
Antisocial behavior	-.117***	.029	-.070	-.065*	.066	.005	-.238***	-.191***	-.229***	-.250***	-.218***	-.250***	-.037	.045	-.025
Negative marijuana-related consequences	-.009	.082	-.033	-.145**	-.014	-.064	-.109*	-.156	-.060	-.235***	-.022	-.175*	-.090	-.169*	-.037
Marijuana frequency	.082**	.124*	.045	.067*	.101	.063	-.071*	.029	-.032	-.116***	-.116*	-.138***	.013	.106*	.038
Marijuana quantity	-.079	-.022	-.029	-.027	-.031	.062	-.162***	-.099	-.068	-.101*	-.031	-.051	.040	.005	.014
Rumination	.124***	-.022	-.100**	-.209***	-.332***	-.214***	-.110***	-.248***	-.217***	-.269***	-.263***	-.241***	-.577***	-.643***	-.563***
Happiness	.133***	.057	.076*	.301***	.334***	.270***	.196***	.165**	.234***	.297***	.250***	.223***	.421***	.392***	.402***

USA = United States; Arg = Argentina; Sp = Spain.

*p < .05;

**p < .01;

***p < .001.

In order to determine if personality dimensions were related differentially to distinct criterion variables across countries, the absolute value of the differences in the magnitude of the correlations for pairs of countries was computed and is presented in Table 7. As the statistical tests of these differences can be oversensitive to small differences when including differences in sample sizes across countries, attention was paid to the magnitude of these differences. The average difference in correlations was .070 (*SD* = .055) across 330 possible comparisons. The results were interpreted using the following: differences <1 *SD* were small, differences between 1 *SD* and 2 *SD* were medium, those between 2 *SD* and 3 *SD* were large, and any over 3 *SD* were substantial. Results presented in Table 7 showed that large or medium size correlation differences across countries were found between conscientiousness and some health outcomes (i.e., anger, mania, anxiety, somatic distress, suicidal ideation and memory) (higher correlations in US than in Argentina or Spain), and also between low agreeableness and low conscientiousness with drug outcomes (higher correlations in US or Spain than in Argentina).

**Table 7:** Absolute value of the correlation differences across countries between the five dimensions of the BFPTSQ and the criterion variables.

	Openness			Extraversion			Agreeableness			Conscientiousness			Emotional Stability		
	USA-Arg	USA-Sp	Arg-Sp	USA-Arg	USA-Sp	Arg-Sp	USA-Arg	USA-Sp	Arg-Sp	USA-Arg	USA-Sp	Arg-Sp	USA-Arg	USA-Sp	Arg-Sp
Depression	.004	.023	.019	.043	.059	.016	.061	.050	.011	.108	.116	.008	.003	.*141*	.*144*
Anger	.003	.044	.047	.009	.104	.113	.076	.008	.068	.*143*	**.215**	.072	.007	.045	.052
Mania	.*142*	.086	.056	.*136*	.117	.019	.122	.*161*	.039	**.204**	**.257**	.053	.093	.103	.010
Anxiety	.041	.056	.097	.*144*	.*172*	.028	.044	.030	.014	**.188**	.*148*	.040	.013	.*143*	.*130*
Somatic distress	.034	.042	.076	.*170*	.103	.067	.012	.022	.034	.*140*	**.197**	.057	.032	.111	.*143*
Suicidal ideation	.*140*	.011	.*129*	.118	.091	.027	.083	.054	.029	**.313**	.111	**.202**	.063	.050	.113
Psychosis	.*148*	.*147*	.001	.109	.111	.002	.113	.021	.092	.*165*	.093	.072	.065	.031	.034
Sleep disturbance	.010	.013	.003	.064	.018	.046	.033	.019	.014	.084	.*162*	.078	.002	.038	.036
Memory	.045	.039	.006	.035	.057	.022	.114	.078	.036	**.195**	.*150*	.045	.036	.120	.084
Repetitive thoughts	.*159*	.033	.*126*	.009	.045	.036	.057	.013	.044	.*129*	.065	.064	.054	.052	.106
Dissociation	.073	.025	.098	.006	.035	.029	.069	.004	.073	.*128*	.117	.011	.013	.068	.081
Personality functioning	.014	.011	.003	.007	.092	.099	.033	.041	.008	.094	.*126*	.032	.048	.086	.*134*
Alcohol use	.105	.054	.051	.008	.118	.110	.130	.022	.*152*	**.187**	.003	**.184**	.116	.019	.097
Tobacco use	.110	.111	.001	.108	.*150*	.042	**.267**	.061	**.206**	.*174*	.*137*	.037	.083	.004	.079
Illicit drug use	.030	.031	.001	.104	.000	.104	.115	.016	.*131*	.109	.068	.041	.023	.054	.077
Total mental health score	.088	.010	.078	.065	.092	.027	.045	.010	.055	.*177*	.*164*	.013	.077	.045	.122
Antisocial behavior	.*146*	.047	.099	.*131*	.070	.061	.047	.009	.038	.032	.000	.032	.082	.012	.070
Negative marijuana-related consequences	.091	.024	.115	.*131*	.081	.050	.047	.049	.096	**.213**	.060	.*153*	.079	.053	.*132*
Marijuana frequency	.042	.037	.079	.034	.004	.038	.100	.039	.061	.000	.022	.022	.093	.025	.068
Marijuana quantity	.057	.050	.007	.004	.089	.093	.063	.094	.031	.070	.050	.020	.035	.026	.009
Rumination	.*146*	**.224**	.078	.123	.005	.118	.*138*	.107	.031	.006	.028	.022	.066	.014	.080
Happiness	.076	.057	.019	.033	.031	.064	.031	.038	.069	.047	.074	.027	.029	.019	.010

Medium correlation differences are shown in *italics* (.125 < *rdiff* < .180), large differences are in **bold** (.180 < *rdiff* < .235), and substantial difference are in **bold and underlined** (*rdiff* > .235).

## Discussion

The present study examined different sources of validity of the English and Spanish versions of the BFPTSQ [8,17] in college students from US, Argentina and Spain. Specifically, we examined whether the BFPTSQ was invariant across two Spanish-speaking populations (Spain and Argentina), across languages (Spanish and English) and across gender. The criterion-related validity was examined via associations among the five BFPTSQ domains and a large set of psychological constructs (i.e., psychopathology, antisocial behavior, marijuana use and negative marijuana-related consequences, rumination and happiness) in the full sample as well as within each country.

### Evidence for internal structure validity

Not surprisingly, when considering previous work that have examined complex structures such as the Big Five [8,17,39], the factor analysis results for the whole sample suggested that ESEM provided a better data fit than the ICM-CFA. Thus, the fact that ESEM allows for all possible factor loadings appears to better approximate the true model than the ICM-CFA. The present study included 125 statistically significant cross-loadings of the 250 possible factor loadings (i.e., 50%). The inclusion of cross-loadings affected the intercorrelations among the personality dimensions as it was shown in Table 4. In the ICM-CFA, cross-loadings were set at 0, and the factor correlations were vastly inflated as this is how these cross-loadings can be represented [8,39]. However, the ESEM not only provides factor correlations that probably come closer to the true population parameters, but also supports the discriminant validity among the Big Five traits as measured by the BFPTSQ [8].

Our findings also indicated an improved model fit when correlated uniquenesses were allowed. Despite including the correlated uniquenesses between the items that were reversed-coded within the same factor or shared the same words being conceptually defensible and increasing the model’s fit, they also inevitably reduced the size of the factor loadings as factors had less variance left to explain. This was salient for the openness factor, which allowed seven correlated uniquenesses and provided lower factor loadings. However, it was noteworthy that the ESEM model’s fit was acceptable even when correlated uniquenesses were allowed, but was still far from excellent according to the typical criteria suggested for practical fit indices [51]. Morin et al. [45] noted that the adequacy of these typical criteria has yet to be demonstrated with ESEM.

All the items presented significant factor loadings on their intended target factor, except items 31 (“is not really interested in different cultures, their customs and values”) and 41 (“has few artistic interests”) of the openness factor (in both the whole sample and the Spanish- and English-speaking samples). A previous study conducted with a general Spanish population sample indicated the primary factor loadings of items 31 and 41 respectively on the openness factors of .38 and .58. The factor loadings for French-speaking [8] and Spanish adolescents [52] were also low. Taken together, and considering that the wording of the items is simple (which arguably implies fewer translation/adaptation problems), these findings suggest that they may not be that suitable for specific populations (i.e., adolescents and young adults) compared to general or adult populations. The rewording, or even the elimination, of these items should be considered in future research, chiefly because the BFPTSQ was developed to supply a useful valid measure for longitudinally assessing personality dimensions across development (i.e., from adolescence to adulthood).

With the extraversion dimension, all the items showed salient factor loadings on their intended factor (i.e., > .30), except for item 42 (“likes exciting activities that provide thrills”). This sensation seeking-related item had a factor loading of .27 on extraversion, and a factor loading of .38 on openness. Previous studies have indicated that sensation seeking tends to be openness-related [53,54].

All the emotional stability and conscientiousness items showed salient factor loadings on their intended factor, but some items in the agreeableness dimension also cross-loaded on the openness factor. As expected, the reverse-coded items that indicated antagonism or low agreeableness loaded on the agreeableness factor, while the positively worded items of this domain cross-loaded on the openness factor. Future revisions of the scale should consider these findings, and the fact that the use of positively worded items and reversed forms in the same scale (e.g., agreeableness) to reduce response bias has been questioned. Suárez-Alvarez et al. [55] illustrated this point and found that this common practice jeopardizes a measure’s unidimensionality by adding secondary sources of variance, and also reduces its reliability.

### Sources of structure validity across groups

In line with previous research, our findings supported the measurement invariance of the BFPTSQ across gender groups [8]. The present findings extend these results by suggesting that the BFPTSQ is also invariant across Spanish-speaking countries. This is a key milestone in cross-cultural research as comparisons between cultures/countries are not valid unless measurement invariance is met [56,57]. Of all the possible comparisons based on CFI changes (in intercepts, factor loadings, uniquenesses, factor variances/covariances, factor latent means and correlated uniquenesses among groups), only four differences were found in the intercepts of the Spanish and Argentinian students. Compared to the Spanish students, the Argentinian ones scored higher for three agreeableness items, which cover the facets of compliance (13r and 38r) and cooperation (43), and also for one conscientiousness item, which covers the facet of deliberation (49r).

Our measurement invariance results across languages (i.e., Spanish and English) revealed that all the agreeableness items loaded primarily on their intended factor in the Spanish-speaking sample. However, in the English-speaking sample, the positively-worded agreeableness items had similar factor loadings on the agreeableness and openness factors as it was shown in Table 3. Having empirically tested the magnitude of these differences, only one difference was found in a primary factor loading: item 20r (“I see myself as someone who worries a lot about many things”). Despite the factor loading of this item being higher in the English-speaking sample than in the Spanish-speaking one, the factor loading was salient and significant in both samples. This finding indicated that it adequately represented its dimension in both groups.

The addition of constraints between intercepts only led to a few noninvariant intercepts across languages. Spanish speakers tended to obtain higher scores than English-speakers for one conscientiousness item tapping the self-discipline facet (4) and for two openness items tapping the intellectual inquisitiveness facet (11 and 36). Compared to the Spanish-speaking participants, English speakers scored higher for: a) two extraversion items tapping the expressiveness (22r) and sociability (32r) facets; b) one for the agreeableness item tapping the compassion facet (28r); c) one conscientiousness item tapping the order facet (9r); and d) one emotional stability item tapping the worry facet (20r). Although a few intercepts and noninvariant loadings were observed across groups based on CFI differences, the RMSEA differences still suggested that the model was completely invariant across languages. The noninvariance of some intercepts and loadings was based mainly on the proposed typical criteria of changes in the fit indices. Nevertheless, these criteria are rough guidelines [42] and some researchers have questioned their validity [58,59], especially for ESEM [45]. Hence rejecting the hypothesis of the invariance of complex models with several items based simply on these typical criteria might not be constructive. Taken together, the results herein obtained suggest that it can be reasonably assumed that the BFPTSQ factor structure offers acceptable measurement invariance across languages.

### Criterion-related validity

The present study aimed to examine the association between the BFPTSQ scores with a wide diversity of outcome variables by particularly focusing on substance use (or substance-related variables) and poor mental health outcomes. As already noted, these behaviors are highly prevalent in college students around the world and a valid, yet brief, version will most likely facilitate both cross-cultural studies and routine interventions to detect students at high risk of developing substance use and/or mental health problems. As in previous studies, low emotional stability, low agreeableness, low conscientiousness and low extraversion were related to mental health outcomes [2,60]. Internalizing symptomatology (i.e., depression, anxiety and somatic distress) was related mainly to low emotional stability [2,60], while antisocial behavior was related to low agreeableness and low conscientiousness in all three countries [33,61].

Other externalizing behaviors, such as drug use, also showed significant correlations with low agreeableness and low conscientiousness, as in previous meta-analysis [2,61], at least in the USA and Spain. The association of disinhibition domains with drug outcomes was less consistent in Argentina and, consequently, some differences in the magnitude of the correlations arose across countries (i.e., medium-size difference in correlations between low conscientiousness and low agreeableness with alcohol use and tobacco use, respectively, in the USA and Spain compared to Argentina). The correlations previously reported between marijuana-related outcomes and conscientiousness/agreeableness [62–65] were only replicated clearly in the USA sample. When we calculated the absolute value of the correlation differences across countries, the only large difference found (i.e., that was between 2 *SD* and 3 *SD* above the average difference in the magnitude of the correlations) was between conscientiousness and marijuana-related problems in the USA and Argentina. Lack of a significant negative association between marijuana-related problems and conscientiousness in the Argentinean sample was somewhat unexpected, as in the case of low conscientiousness and low agreeableness with alcohol, tobacco and illicit drug use. Future research should replicate this finding to know if the disinhibition-related domains assessed within the FFM framework could influence drug outcomes in Argentinian college students.

In line with previous studies conducted using the BFPTSQ and other measures to assess the FFM, happiness was mainly related to both emotional stability (or low neuroticism) and extraversion [17,66,67], while rumination was related chiefly to conscientiousness, emotional stability, agreeableness and extraversion [37].

### Limitations and conclusions

Our research is not without its limitations. First, even though the BFPTSQ’s psychometric properties have been previously explored in a Spanish population [17], this is the first time that the English version structure has been tested. Based on some of our results (i.e., the nonsignificant factor loadings of items 31 and 41 on the openness factor, or the cross-loadings between the openness and agreeableness items), replication studies are needed before the questionnaire can be modified (i.e., remove or substitute items). Our sample comprised of university students, and future research should examine the reliability and construct validity of the English questionnaire version in both adolescent and general adult populations. Finally, our work explored evidence for criterion validity with a limited number of outcomes (i.e., psychopathology, antisocial behavior, marijuana-related outcomes, rumination, and happiness). Previous work has found an association between personality and a wide range of other health-related behaviors (e.g., work and educational outcomes [5,68]). Future research that employs the BFPTSQ could benefit from including more criterion variables.

Despite its limitations, the present research supports the BFPTSQ’s factor validity, the reasonable invariance of the measure across genders, across two Spanish-speaking populations, and between Spanish and English speakers. It also evidences the scales’ reliability and criterion validity (associations with distinct health outcomes). Taken together, our results suggest the BFPTSQ is a useful short measure for assessing the FFM broad domains between English and Spanish speakers, at least for young adults from the USA, Argentina and Spain.

## Supporting information

S1 Table Correspondence of the English / Spanish version of the BFI with the items of the BFPTSQ. A) English, B) Spanish. In bold are marked the items that are identical in both questionnaires. The items can also be found in: Benet-Martinez, V., & John, O. P. (1998). Los Cinco Grandes across cultures and ethnic groups: Multitrait multimethod analyses of the Big Five in Spanish and English. *Journal of Personality and Social Psychology*, *75*, 729–750; and Morizot, J. (2014). Construct validity of adolescents’ self-reported big five personality traits: importance of conceptual breadth and initial validation of a short measure. *Assessment*, *21*, 580–606.
(DOCX)

S2 Table Standardized Factor Loadings from the Exploratory Structural Equation Model of the BFPTSQ in Argentinian and Spanish Samples (M2b). The columns “Argentina” and “Spain” correspond to the factor loadings of the M2b model (see Table 2). Bold denotes all the significant factor loadings (the 99% CI does not cross zero). λ = factor loadings; δ = uniquenesses.
(DOCX)
