# Supplementary material for: Cross-cultural examination of the Big Five Personality Trait Short Questionnaire: Measurement invariance testing and associations with mental health
Source: PLoS One. 2019 Dec 17;14(12):e0226223. doi: 10.1371/journal.pone.0226223 (PMC6917272; doi:10.1371/journal.pone.0226223)
Supplement: S1 Table — A) English, B) Spanish. In bold are marked the items that are identical in both questionnaires. The items can also be found in: Benet-Martinez, V., & John, O. P. (1998). Los Cinco Grandes across cultures and ethnic groups: Multitrait multimethod analyses of the Big Five in Spanish and English. Journal of Personality and Social Psychology, 75, 729–750; and Morizot, J. (2014). Construct validity of adolescents’ self-reported big five personality traits: importance of conceptual breadth and initial validation of a short measure. Assessment, 21, 580–606. (DOCX) [file pone.0226223.s001.docx]

S1 Table. Correspondence of the English / Spanish version of the BFI with the items of the BFPTSQ

1. English

| **Trait** | **BFI** | **BFPTSQ** |
| --- | --- | --- |
| Extraversion | 1. is talkative | 2. likes to talk, express his/her opinion |
|  | 6. is reserved | 7. is reserved or shy, has difficulty approaching others |
|  | 11. is full of energy | 12. is full of energy, likes to always be active |
|  | 16. generates a lot of enthusiasm | - |
|  | 21. tends to be quiet | 22. is rather quiet, does not talk a lot |
|  | 26. has an assertive personality | 27. shows self-confidence, is able to assert himself/herself |
|  | 31. is sometimes shy, inhibited | 32. Is timid, shy |
|  | 36. is outgoing, sociable | 37. is extraverted, sociable |
|  |  | 42. likes exciting activities, which provides thrills. |
|  |  | 47. has a tendency to laugh and have fun easily |
|  |  | 17. is a leader, capable of convincing others |
| Agreeableness | 2. tends to find fault with others | 3. has a tendency to criticize others |
|  | 7. is helpful and unselfish with others | 8. is helpful and generous with others |
|  | 12. starts quarrels with others | 13. provokes quarrels or arguments with others |
|  | 17. has a forgiving nature | 18. is lenient, forgives easily |
|  | 22. is generally trusting | 23. generally trust others |
|  | 27. can be cold and aloof | 28. can be distant and cold towards others |
|  | **32. is considerate and kind to almost everyone** | **33. is considerate and kind to almost everyone** |
|  | 37. is sometimes rude to others | 38. can sometimes be rude and mean towards others |
|  | **42. likes to cooperate with others** | **43. likes to cooperate with others** |
|  |  | 48. Can deceive and manipulate people to get what he/she want |
| Conscientiousness | 3. does a thorough job | 4. works conscientiously does the things he/she has to do well |
|  | 8. can be somewhat careless | 9. can be little careless and negligent |
|  | 13. is a reliable worker | 14. is a reliable student/worker, who can be counted on |
|  | 18. tends to be disorganized | 19. has a tendency to disorganized, messy |
|  | 23. tends to be lazy | 24. has a tendency to be lazy |
|  | 28. perseveres until die task is finished | 29. perseveres until the task at hand is completed |
|  | 33. does things efficiently | 34. does things efficiently, works well and quickly |
|  | 38. makes plans and follows through with them | 39. plans things that need to be done and follows through the plans |
|  | 43. is easily distracted | 44. is easily distracted, has difficulty remaining attentive |
|  |  | 49. can do things impulsively without thinking about the consequences |
| Emotional Stability | 4. is depressed, blue | 5. has a tendency to be easily depressed, sad |
|  | 9. is relaxed, handles stress well | 10. is generally relaxed, handles stress well |
|  | 14. can be tense | 15. can be tense, stressed out |
|  | 19. worries a lot | 20. worries a lot about many things |
|  | **24. is emotionally stable, not easily upset** | **25. is emotionally stable, not easily upset** |
|  | **29. can be moody** | **30. can be moody** |
|  | 34. remains calm in tense situations | 35. stays calm in tense or stressful situations |
|  | 39. gets nervous easily | 40. can easily become nervous |
|  |  | 45. has a tendency to feel inferior to others |
|  |  | 50. has a tendency to be easily irritated |
| Openness to Experience | 5. is original, comes up with new ideas | 1. is original, often has new ideas |
|  | **10. is curious about many different things** | **6. is curious about many different things** |
|  | 15. is ingenious, a deep thinker | 11. is ingenious, reflects a lot |
|  | 20. has an active imagination | 16. has a lot of imagination |
|  | 25. is inventive | 21. is inventive, creative |
|  | 30. values artistic, aesthetic experiences | 26. likes artistic or aesthetics experiences |
|  | 35. prefers work that is routine | - |
|  | 40. likes to reflect, play with ideas | 36. likes to reflect, tries to understand complex things |
|  | **41. has few artistic interests** | **41. has few artistic interest** |
|  | 44. is sophisticated in art, music, or literature | 46. is sophisticated when it comes to art, music or literature |
|  |  | 31. is not really interested in different cultures, their customs and values |

1. Spanish.

| **Rasgo** | **BFI** | **BFPTSQ** |
| --- | --- | --- |
| Extraversión | 1. es bien hablador | 2. Le gusta hablar, expresa sus opiniones |
|  | 6. es reservado | 7. Es reservado/a o tímido/a, tiene dificultad para acercarse a los demás |
|  | 11. está lleno de energía | 12. está lleno/a de energía, le gusta estar siempre activo/a |
|  | 16. irradia entusiasmo | - |
|  | 21. tiende a ser callado | 22. es más bien reservado/a, no habla mucho |
|  | 26. es asertivo | 27. muestra autoconfianza, es capaz de actuar decisivamente |
|  | 31. es a veces tímido, inhibido | 32. es tímido/a |
|  | **36. Es extrovertido, sociable** | **37. es extravertido/a, sociable** |
|  |  | 42. le gustan las actividades estimulantes, que proporcionen sensaciones fuertes |
|  |  | 47. tiende a reír y divertirse con facilidad |
|  |  | 17. es un/a líder, capaz de convencer a los demás |
| Amabilidad | 2. tiende a ser criticón | 3. tiende a criticar a los demás |
|  | 7. es generoso y ayuda a los demás | 8. ayuda y es generoso/a con los demás |
|  | 12. inicia disputas con los demás | 13. provoca riñas o discusiones con los demás |
|  | 17. es indulgente, no le cuesta personar | 18. es benévolo/a, perdona fácilmente |
|  | 22. es generalmente confiado | 23. generalmente confía en los demás |
|  | 27. es a veces frío y distante | 28. puede ser distante y frío/a con los demás |
|  | 32. es considerado y amable con casi todos | 33. es considerado/a y amable con casi todo el mundo |
|  | 37. es a veces maleducado con los demás | 38. puede ser grosero/a o desagradable con los demás |
|  | 42. le gusta cooperar con los demás | 43. le gusta colaborar con los demás |
|  |  | 48. puede engañar y manipular a la gente para conseguir lo que quiere |
| Responsabilidad | 3. es minucioso en el trabajo | 4. trabaja a conciencia, hace bien las cosas que debe hacer |
|  | 8. puede a veces ser algo descuidado | 9. puede ser un poco descuidado/a y poco aplicado/a |
|  | 13. es un trabajador cumplidor, de confianza | 14. es un/a estudiante – trabajador/a fiable, con quien se puede contar |
|  | 18. tiende a ser desorganizado | 19. tiende a se desorganizado/a, descuidado/a |
|  | 23. tiende a ser flojo, vago | 24. tiende a ser perezoso/a |
|  | 28. persevera hasta terminar el trabajo | 29. no deja una tarea hasta que está acabada |
|  | 33. hace las cosas de manera eficiente | 34. hace las cosas eficientemente, trabaja bien y con rapidez |
|  | 38. hace planes y los sigue cuidadosamente | 39. planea las cosas que hay que hacer y las realiza de principio a fin |
|  | 43. se distrae con facilidad | 44. se distrae con facilidad, le cuesta mantener la atención |
|  |  | 49. puede hacer cosas impulsivamente sin pensar en las consecuencias |
| Estabilidad Emocional | 4. es depresivo, melancólico | 5. tiende a estar deprimido/a con facilidad, triste |
|  | 9. es calmado, controla bien el estrés | 10. generalmente está relajado/a, maneja bien el estrés |
|  | 14. con frecuencia se pone tenso | 15. puede estar tenso/a, estresado/a |
|  | 19. se preocupa mucho por las cosas | 20. se preocupa mucho por la mayoría de las cosas |
|  | 24. es emocionalmente estable, difícil de alterar | 25. es estable emocionalmente, no se disgusta fácilmente |
|  | 29. es temperamental, de humor cambiante | 30. puede tener cambios frecuentes de humor |
|  | 34. mantiene la calma en situaciones difíciles | 35. permanece calmado/a en situaciones tensas o estresante |
|  | 39. se pone nervioso con facilidad | 40. puede ponerse nervioso/a fácilmente |
|  |  | 45. tiende a sentirse inferior a los demás |
|  |  | 50. tiende a irritarse fácilmente |
| Apertura a la Experiencia | 5. es original, se le ocurren ideas nuevas | 1. es original, a menudo tiene ideas nuevas |
|  | 10. tiene intereses muy diversos | 6. es curioso/a acerca de muchas cosas diferentes |
|  | 15. es ingenioso, analítico | 11. es ingenioso/a, reflexiona mucho |
|  | 20. tiene una imaginación activa | 16. tiene mucha imaginación |
|  | 25. es inventivo | 21. tiene imaginación, creative/a |
|  | 30. valora lo artístico, estético | 26. le gustan las experiencias artísticas o estéticas |
|  | 35. prefiere trabajos que son rutinarios | - |
|  | 40. le gusta reflexionar, jugar con las ideas | 36. le gusta reflexionar, intenta entender cosas complicadas |
|  | 41. tiene pocos intereses artísticos | 41. tiene pocos intereses artísticos |
|  | 44. es educado en arte, música o literatura | 46. es sofisticado/a cuando se trata de arte, música o literatura |
|  |  | 31. no está muy interesado/a en otras culturas, sus costumbre y valores |

In bold are marked the items that are identical in both questionnaires. The items can also be found in: Benet-Martinez, V., & John, O. P. (1998). Los Cinco Grandes across cultures and ethnic groups: Multitrait multimethod analyses of the Big Five in Spanish and English. *Journal of Personality and Social Psychology, 75*, 729-750; and Morizot, J. (2014). Construct validity of adolescents’ self-reported big five personality traits: importance of conceptual breadth and initial validation of a short measure. *Assessment*, *21*, 580–606.
