# Supplementary material for: Cross-cultural examination of the Big Five Personality Trait Short Questionnaire: Measurement invariance testing and associations with mental health
Source: PLoS One. 2019 Dec 17;14(12):e0226223. doi: 10.1371/journal.pone.0226223 (PMC6917272; doi:10.1371/journal.pone.0226223)
Supplement: S2 Table — The columns “Argentina” and “Spain” correspond to the factor loadings of the M2b model (see Table 2). Bold denotes all the significant factor loadings (the 99% CI does not cross zero). λ = factor loadings; δ = uniquenesses. (DOCX) [file pone.0226223.s002.docx]

**S2 Table. Standardized Factor Loadings from the Exploratory Structural Equation Model of the BFPTSQ in Argentinian and Spanish Samples (M2b)**

|  | **λ Openness** | | **λ Extraversion** | | **λ Agreeableness** | | **λ Conscientiousness** | | **λ Emotional stability** | | **δ** | **δ** |
| --- | --- | --- | --- | --- | --- | --- | --- | --- | --- | --- | --- | --- |
| **Item** | **Argentina** | **Spain** | **Argentina** | **Spain** | **Argentina** | **Spain** | **Argentina** | **Spain** | **Argentina** | **Spain** | **Argentina** | **Spain** |
| 1 | **.519** | **.421** | **.253** | **.259** | .024 | .039 | .090 | .083 | .104 | .086 | .572 | .656 |
| 6 | **.456** | **.409** | .035 | **.137** | **.226** | **.239** | .035 | .003 | -.095 | -.029 | .686 | .693 |
| 11 | **.545** | **.506** | .098 | **.122** | .066 | .050 | **.171** | **.109** | .078 | **.142** | .592 | .617 |
| 16 | **.529** | **.426** | .095 | **.165** | .066 | .037 | .036 | -.031 | -.100 | **.103** | .659 | .743 |
| 21 | **.501** | **.438** | **.141** | **.172** | .052 | .031 | .067 | .022 | -.065 | **.138** | .673 | .705 |
| 26 | **.394** | **.349** | -.029 | .078 | **.175** | .104 | -.055 | -.012 | .042 | .072 | .796 | .826 |
| 31r | .119 | .032 | .081 | .059 | **.234** | **.262** | .044 | -.072 | -.115 | .069 | .901 | .915 |
| 36 | **.438** | **.399** | .096 | .013 | .028 | **.238** | .165 | **.157** | -.010 | .083 | .734 | .657 |
| 41r | **.261** | .063 | -.017 | .078 | .088 | .128 | .035 | -.002 | .056 | .119 | .910 | .941 |
| 46 | **.279** | **.270** | .028 | .064 | .001 | .039 | .058 | .045 | -.070 | .044 | .907 | .898 |
| 2 | **.152** | **.254** | **.564** | **.541** | .063 | .075 | -.005 | .074 | .044 | -.085 | .600 | .551 |
| 7r | -.107 | **-.223** | **.827** | **.831** | .063 | -.008 | .003 | -.069 | .020 | .060 | .312 | .327 |
| 12 | **.183** | **.263** | **.354** | **.327** | .081 | .107 | .183 | **.220** | .090 | .084 | .710 | .619 |
| 17 | **.404** | **.370** | **.361** | **.426** | **-.230** | **-.258** | **.192** | **.149** | .114 | **.116** | .591 | .559 |
| 22r | **-.274** | **-.287** | **.886** | **.876** | .046 | .074 | -.025 | -.029 | -.005 | -.071 | .224 | .251 |
| 27 | **.362** | **.351** | **.347** | **.326** | -.122 | -.126 | .161 | **.215** | **.338** | **.260** | .535 | .549 |
| 32r | **-.157** | **-.249** | **.837** | **.860** | .011 | -.035 | .068 | **-.081** | .045 | .056 | .288 | .282 |
| 37 | .063 | **.108** | **.825** | **.764** | .059 | .026 | -.082 | -.014 | .008 | -.069 | .287 | .385 |
| 42 | **.296** | **.296** | **.202** | **.253** | -.051 | .024 | -.197 | **-.154** | -.013 | -.013 | .831 | .823 |
| 47 | **.200** | **.267** | **.327** | **.275** | .188 | **.352** | -.105 | .012 | .106 | .038 | .746 | .620 |
| 3r | -.076 | **-.138** | **-.147** | **-.130** | **.328** | **.365** | .068 | .008 | **.178** | **.290** | .817 | .739 |
| 8 | **.299** | **.342** | .043 | .077 | **.573** | **.547** | -.008 | .063 | -.094 | **-.138** | .523 | .477 |
| 13r | -.092 | -.108 | **-.202** | -.074 | **.415** | **.537** | .123 | **.141** | **.207** | **.202** | .701 | .569 |
| 18 | **.219** | **.213** | -.017 | .022 | **.359** | **.361** | -.084 | -.060 | .126 | .021 | .776 | .802 |
| 23 | **.202** | **.169** | **.179** | .102 | **.430** | **.376** | **-.211** | -.113 | .000 | -.012 | .676 | .804 |
| 28r | **-.353** | **-.351** | **.448** | **.262** | **.435** | **.386** | .030 | -.041 | -.065 | .050 | .550 | .705 |
| 33 | .177 | **.233** | .057 | .062 | **.534** | **.556** | -.002 | .058 | -.025 | -.024 | .639 | .546 |
| 38r | **-.223** | **-.164** | **-.161** | **-.107** | **.581** | **.507** | .096 | **.156** | **.183** | **.171** | .539 | .613 |
| 43 | **.281** | **.223** | **.156** | **.163** | **.504** | **.468** | -.056 | .047 | -.077 | -.073 | .574 | .622 |
| 48r | **-.255** | **-.344** | -.101 | -.064 | **.466** | **.596** | **.198** | .077 | .108 | -.062 | .652 | .588 |
| 4 | **.351** | **.273** | -.036 | .013 | .084 | .073 | **.515** | **.560** | **-.140** | -.078 | .573 | .546 |
| 9r | **-.259** | **-.330** | .052 | -.027 | -.029 | -.081 | **.557** | **.656** | -.035 | -.024 | .651 | .545 |
| 14 | **.298** | **.242** | .045 | .050 | **.213** | **.236** | **.290** | **.430** | -.083 | **-.160** | .723 | .595 |
| 19r | -.164 | **-.270** | -.053 | -.052 | -.072 | .040 | **.595** | **.614** | .053 | -.059 | .624 | .601 |
| 24r | -.060 | **-.300** | **.163** | **.121** | -.006 | -.031 | **.469** | **.540** | .012 | **.117** | .735 | .595 |
| 29 | .062 | **.212** | -.037 | -.048 | .057 | -.044 | **.513** | **.596** | -.129 | -.074 | .735 | .611 |
| 34 | **.317** | **.266** | .071 | .080 | .103 | .065 | **.446** | **.534** | -.109 | -.001 | .633 | .539 |
| 39 | .160 | **.151** | -.002 | -.016 | -.131 | -.017 | **.539** | **.622** | -.059 | **-.141** | .691 | .598 |
| 44r | -.112 | **-.240** | .077 | .048 | -.077 | **-.155** | **.428** | **.559** | **.241** | **.186** | .698 | .605 |
| 49r | -.090 | **-.205** | **-.184** | **-.192** | **.198** | **.258** | **.276** | **.282** | **.319** | **.277** | .693 | .663 |
| 5r | -.056 | -.092 | **.201** | **.187** | .055 | .081 | .148 | .062 | **.531** | **.514** | .556 | .613 |
| 10 | **.305** | **.375** | .022 | **-.095** | .003 | -.026 | **-.231** | **-.131** | **.695** | **.630** | .476 | .517 |
| 15r | **-.181** | -.077 | .042 | .017 | .092 | .018 | -.104 | **-.100** | **.560** | **.657** | .624 | .577 |
| 20r | **-.172** | **-.249** | .077 | .024 | **-.161** | **-.285** | **-.174** | **-.208** | **.590** | **.475** | .616 | .623 |
| 25 | **.242** | **.244** | -.042 | .021 | .081 | .011 | .008 | .047 | **.715** | **.562** | .420 | .590 |
| 30r | **-.135** | **-.155** | .021 | .006 | **.178** | **.147** | .105 | .061 | **.642** | **.621** | .428 | .514 |
| 35 | **.253** | **.397** | -.058 | -.071 | .021 | .021 | .022 | .017 | **.565** | **.575** | .633 | .509 |
| 40r | **-.147** | .008 | .067 | -.017 | .001 | -.034 | -.006 | -.003 | **.706** | **.764** | .453 | .432 |
| 45r | .010 | -.021 | **.234** | **.295** | **-.193** | **-.136** | .129 | **.211** | **.559** | **.451** | .574 | .588 |
| 50r | -.099 | -.058 | -.045 | -.063 | **.233** | **.278** | .114 | .051 | **.637** | **.662** | .414 | .397 |

The columns “Argentina” and “Spain” correspond to the factor loadings of the M2b model (see Table 2). Bold denotes all the significant factor loadings (the 99% CI does not cross zero). λ = factor loadings; δ = uniquenesses.
